# A Review of the Polymer for Cryogenic Application: Methods, Mechanisms and Perspectives

**DOI:** 10.3390/polym13030320

**Published:** 2021-01-20

**Authors:** Duo Chen, Juanzi Li, Yuhuan Yuan, Chang Gao, Yunguang Cui, Shichao Li, Xin Liu, Hongyu Wang, Cong Peng, Zhanjun Wu

**Affiliations:** 1 Faculty of Mechanical Engineering Materials and Energy, School of Materials Science and Engineering, Dalian University of Technology, Dalian 116024, China; chenduo93@163.com (D.C.); jzl.1101.xpu@gmail.com (J.L.); yuanyuhuan0000@163.com (Y.Y.); rainy.gao723@dlut.edu.cn (C.G.); 2 State Key Laboratory of Structural Analysis for Industrial Equipment, Faculty of Vehicle Engineering and Mechanics, School of Aeronautics and Astronautics, Dalian University of Technology, Dalian 116024, China; cyg1363956827@163.com (Y.C.); liuxindlut@dlut.edu.cn (X.L.); why2216@mail.dlut.edu.cn (H.W.); dalianpcpc@126.com (C.P.)

**Keywords:** polymer, modification, cryogenic properties, liquid oxygen compatibility

## Abstract

Recently, the application of polymer-based composites at cryogenic conditions has become a hot topic, especially in aerospace fields. At cryogenic temperature, the polymer becomes more brittle, and the adverse effect of thermal stress induced by temperature is more remarkable. In this paper, the research development of thermoset and thermoplastic polymers for cryogenic applications are all reviewed. This review considers the literature concerning: (a) the cryogenic performance of modified thermoset polymers and the improving mechanisms of the reported modification methods; (b) the cryogenic application potential of some commercial thermoplastic polymers and the cryogenic performance of modified thermoplastic polymers; (c) the recent advance in the use of polymer for special cryogenic environment-liquid oxygen. This paper provides a comprehensive overview of the research development of the polymer for cryogenic application. Moreover, future research directions have been proposed to facilitate its practical applications in aerospace.

## 1. Introduction

Cryogenic liquids, such as liquid helium (LHe,4 K, liquid hydrogen (LH_2_,20 K), Liquid nitrogen (LN_2_,77 K), liquid oxygen (LOX,90 K), Liquid methane (LMG,112 K) play an important role in aerospace, superconductivity, energy and medical fields [1,2,3]. Due to the light weight, high specific strength, excellent corrosion resistance, good anti-fatigue performance and designability of the polymer-based composites, the research and application of polymeric matrix composites in the cryogenic fields has become a hot topic, especially in aerospace [4,5,6,7,8,9,10]. Polymer-based composites materials offer significant advantages in space applications with weight reduction of 25% to 35% and cost savings of near 50% [4,5,7]. The current cost to send 1 Kg of payload into geosynchronous transfer orbit using the SpaceX Falcon launcher is approximately $7500 [8]. Thus, paying more attention to polymer matrix composites for cryogenic application is an interesting and meaningful work.

In cryogenic regions, the cryogenic tanks of launch vehicles are a particularly difficult application for polymer-based composite materials [6,9]. Not only must laminates used in these applications be very structurally efficient, but they must also contain extremely volatile cryogenic propellants (such as LH_2_ and LOX) [9]. A brief introduction of composites cryogenic tank research process in some projects is shown below. In 1987, McDonnell Douglas Corporation (MDC) completed the first LH_2_ composite cryogenic tank for a single stage to orbit (SSTO) spaceplane (X-30). This tank was fabricated using the thermoplastic graphite-reinforced material with a high glass transition temperature [5,7]. In 1994, the LH_2_ composite cryogenic tank of DC-XA was fabricated in two halves using a carbon/epoxy material by Douglas Aircraft Company [8]. At the same time, Lockheed Martin Space Systems Company build a honeycomb (HC) sandwich structure composites LH_2_ tank of the reusable launch vehicle (RLV) X-33 with the carbon/epoxy prepreg. Subsequently, other reusable launch vehicles such as orbital test vehicle (OTV) X-34 continued to evaluate composite cryogenic tank [11]. In the 2010s, during the composite cryogenic tank technology development (CCTD) project of NASA’s Game Changing Development Program (GCDP), polymeric matrix composite cryogenic tanks with diameters of 2.4 m, 5.5 m and 10 m were built by the Boeing Company [5]. In 2014, during the Cryogenic Hypersonic Advanced Tank Technologies (CHATT) of the European Commission’s Seventh Framework Program, a polymeric matrix composites cryogenic tank with a Polyethylene liner was manufactured [6]. In 2016, a 12 m diameter polymeric matrix composites LOX tank was fabricated by Space Exploration Technologies Corporation (Space X) [12]. This composites LOX tank was the largest cryogenic tank ever produced so far.

Although most of the above projects attempt to adopt mature material systems to reduce technical risks, there are still various problems for lack of enough information about the performance of polymeric composites under cryogenic environment [3,4,5,6,7,8]. Numerous works on the design and analysis of composite cryogenic tank has intensified since the failure of the X-33 RLV fuel tank, where fuel leakage occurred on the tank wall. The leakage was due to the fractures in the polymer and the high thermal stress caused by the mismatch of coefficient of thermal expansion (CTE) between the polymer and carbon fiber [12,13,14,15,16,17,18]. While, for reusable launch vehicles, the cryogenic performance of polymer-based composites under thermal and mechanical cyclic load is one of the most critical issue. Shindo’s research group has studied the Delamination growth mechanisms and fatigue behavior of fiber reinforced polymer based woven composites under Mode I [19], Mode II [20], Mode III [21] and mixed-mode I/II [22] fatigue loadings at 77 K and 4 K. The fatigue delamination growth rates of fiber reinforced polymer-based woven composites at 77 K and 4 K were lower than those at room temperature. Their interesting and meaningful work can lay a foundation for the design and application of polymer-based composites in cryogenic environment. At cryogenic temperature, when the fiber-reinforced polymer-based composites structure is subjected to combined thermal and mechanical loads, microcracks may initiate at loads well below the ultimate strength of the composite materials [23]. The maximum residual thermal stress in the carbon/epoxy composite is the longitudinal tensile stress in the matrix, which is a potential danger to the safety of composites structure for cryogenic use [24]. Meanwhile, the residual thermal stress has a significant effect on the stress distributions near the crack fronts in the composites at cryogenic temperatures [25].

Therefore, improving the mechanical properties including strength, ductility, impact strength and reducing the CTE of the polymer is desirable in developing high-performance composites for cryogenic engineering applications.

**Figure 1:**
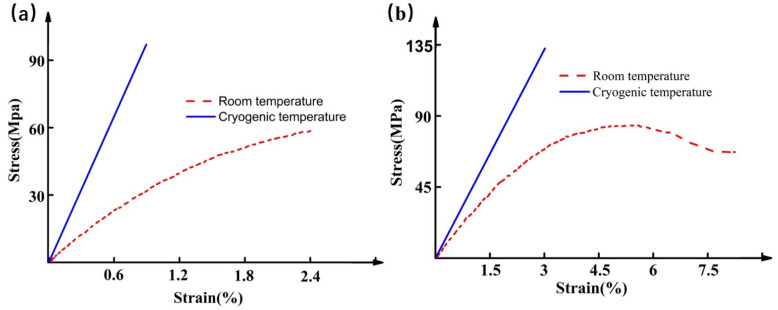
The typical tensile stress-strain behaviors of (**a**) thermoset polymer and (**b**) thermoplastic polymer under room and cryogenic temperature.

The polymers used in composite applications include thermosets and thermoplastics, or blends of the two. In thermoset composites, the matrix starts as low molecular weight material. During the curing process, the resin forms a rigid, high molecular weight structure. This rigidity is caused by the crosslinks (usually covalent bonds) formed among the polymer chains, which give the resin strength and thermal stability. As the crosslinking density (crosslinks per unit volume) increases and chain length decreases, the movement of polymer chains is more constrained and the resin becomes stiffer. This effect is magnified when the resin is exposed to cryogenic temperatures [11,14]. As Figure 1a shows, taking tensile properties as an example, the tensile strength, modulus and fracture strain of thermoset polymers demonstrate a dramatic change. Many works have been done to enhance the toughness and strength of thermoset polymers at room temperature. Very often, these properties can be effectively improved by introducing various toughening agents, such as inorganic fillers, block copolymer, hyperbranched polymer, reactive diluents, reactive liquid rubber, and thermoplastic [17,18,26,27,28,29,30,31,32]. Such research has made remarkable achievements. Thus, it is feasible to improve the mechanical properties of the thermoset polymer at cryogenic temperature by adopting similar methods. Unlike thermoset polymers, the thermoplastic polymer does not crosslink among polymer backbone chains, which gives thermoplastic composites the unique ability to be reprocessed by melting and reconsolidating [33]. In contrast to thermoset polymer, the thermoplastic polymer’s mechanical properties under different environments exhibit a similar variation trend (Figure 1b). To date, due to the limitation of relatively complicated manufacturing process and high cost, no large-scale studies have been performed to investigate the mechanical performance of thermoplastic polymer and its composites under cryogenic temperature [34]. With the development of science and technology, thermoplastic polymers will have a better prospect application in the cryogenic engineering field based on the high toughness, recycle ability.

**Figure 2:**
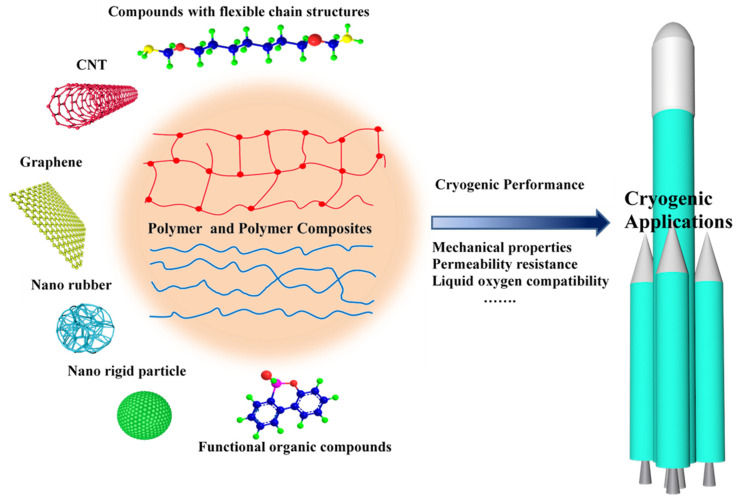
Schematic illustration of the main contents in this review, including the cryogenic performance of thermoset, thermoplastic polymer and the modified polymer by introducing various modifiers, such as graphene, carbon nanotubes, nanoparticle, rubber, compounds with flexible chain structures and functional organic compounds.

Figure 2 provides an overview of the research and development of polymer and modified polymer for cryogenic application. The modification approaches, effects and mechanisms of the modified polymer at cryogenic temperature are all summarized. In this review, as mentioned above, Section 2 and Section 3 present the research and development of thermoset and thermoplastic polymer under cryogenic applications. To the polymer-based composites applied for LOX, besides meeting the physical requirements at cryogenic temperature, the polymer-based composites adopted in LOX systems must also be chemically compatible with LOX. Subsequently, Section 4 presents the recent advances in the use of polymer for liquid oxygen. Finally, Section 5 summarizes the advances made on polymers for cryogenic applications, where we are now, and provides insight into future research in the cryogenic polymer-based composites.

## 2. The Research Development of Thermoset Polymer for Cryogenic Application

Generally, thermoset polymers such as epoxy (EP) resin, phenolic resin, polyester resin, bismaleimide resin, etc., are made of an initial liquid mixture of polyfunctional organic molecules. They gradually react with each other to form a three-dimensional cross-link network [35]. Among these numerous thermoset polymers, EP-based thermosets hold a prominent position for their high adhesion strength, excellent mechanical properties and good processability [27,36]. However, the high cross-link density lowers the fracture toughness and the resistance to crack initiation of the thermosets. This largely restricts their applications in a cryogenic environment [11]. Therefore, toughening of thermoset polymers has become a necessity to ensure the feasibility of these materials for practical applications. These drawbacks can be overcome by decreasing the cross-link density of thermoset resin or incorporating heterogeneous phases with some additives such as rigid inorganic nanomaterial, soft polymers (elastomer, block copolymers, high-performance plastic polymer and hyperbranched polymer) [18,26,27,28,29,30,31,32].

In this section, the research and application development under the cryogenic temperatures of thermoset polymers modified by various components (rigid nanomaterial, thermoplastic, rubber and other compounds) are summarized. Meanwhile, the effects on the cryogenic properties of thermoset polymers modified by changing their cross-link structure are also reviewed.

**Figure 3:**
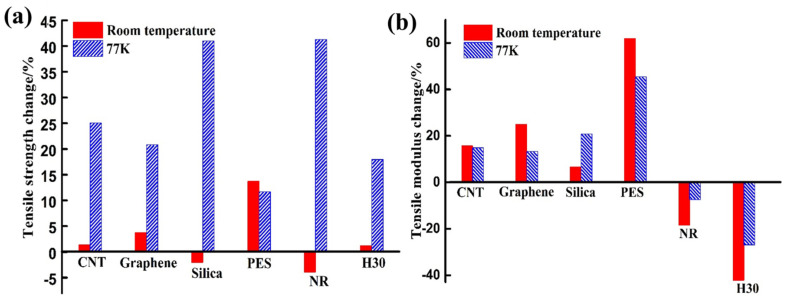
(**a**) The tensile strength and (**b**) tensile modulus change of CNT [37], graphene [40], silica [45], PES [48], NR [52] and H30 [55] modified polymer composites compared with unmodified polymers at room and cryogenic temperature.

Table 1 exhibits previous works on the mechanical properties of thermoset polymers modified by nanomaterial, thermoplastic, rubber and other compounds at the cryogenic temperatures. From this table, the current mechanical level that the modified thermoset polymer can achieve at cryogenic temperature could be clearly realized. As shown in Figure 3, we also summarized the modification effect of different method on tensile properties at different temperatures. Compared with pristine polymers, the increase or decrease in tensile properties of modified polymers at room temperature doesn’t mean that would demonstrate the same variation trend at cryogenic temperatures. Introducing the fillers into polymers, such as CNTs, Graphene and PES, the tensile strength and modulus of polymers at room or cryogenic temperature all could be improved. However, the effects of modification method cannot be predicted. Meanwhile, the research progress about such methods is described in detail below.

### 2.1. Enhancing the Cryogenic Performance of Thermoset Polymer by Introducing the Rigid Nanomaterial

#### 2.1.1. Carbon Nanotubes

Carbon nanotubes (CNTs) have attracted a huge deal of attention owing to their exceptional mechanical and physical properties. High aspect ratio (length/diameter) and low density have made them a promising reinforcing material for polymer matrix composites [55,56,57]. There are some ways to enhance polymer matrix composites with carbon nanotubes (CNTs). Such as mixing CNTs into a polymer matrix, grafting CNTs on the fiber for the matrix-fiber interface region, and inserting paper-formed CNTs to the laminar interface [30,55]. Among these methods, mixing CNTs into the polymer matrix is the most efficient, economical and convenient one. However, weak interfacial bonding between CNTs and polymers leads to poor stress transfer, limiting the full realization of CNTs as reinforcements for polymers [33,57,58,59].

**Figure 4:**
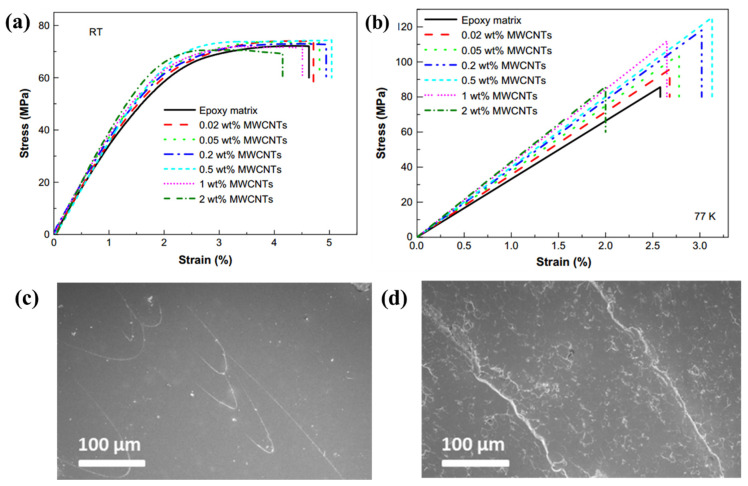
The stress-strain curves of MWCNT/epoxy nanocomposites (**a**) at room temperature and (**b**) at 77 K. Reused with permission from Ref. [37]. Copyright 2009 Elsevier. The SEM images of impact fracture surfaces at 77 K of (**c**) epoxy and (**d**) 0.5 wt CNTs modified epoxy. Reused with permission from Ref. [39]. Copyright 2013 Elsevier.

Chen et al. studied the cryogenic mechanical properties of MWCNTs reinforced EP nanocomposites [37]. The MWCNTs were treated using a mixture of concentrated sulfuric and nitric acids. The acid-treated MWCNTs were dispersed into the epoxy via the ultrasonic technique. The tensile mechanical properties of epoxy nanocomposites containing MWCNTs with weight fractions of 0.02%, 0.05%, 0.2%, 0.5%, 1%, and 2% were tested at room temperature and 77 K (Figure 4a,b). It was found that the tensile strength, toughness and impact strength reached the maximum at cryogenic temperature when the MWCNT content was 0.5 wt%. With the temperature decreasing from room temperature to cryogenic temperature, the big differences of thermal expansion coefficients between EP and the uniformly dispersed MWCNTs led to a thermal contraction of the EP matrix. The contraction generated a stronger MWCNT–EP interfacial bonding. Due to the strong interfacial bonding, the interface is capable of transferring load more effectively from the polymer to the MWCNTs. Thus, the cryogenic mechanical properties of polymers were enhanced by MWCNTs.

The strengthening effects of aminated MWCNTs (MWCNTs-NH_2_) on the cryogenic mechanical performance of EP-based composites were discussed by Jia, et al. [38]. The EP-based composites contain a 30% soft polymer modifier, EP grafted polyurethane (PU), which was synthesized by controlling the reaction between –OH in EP and –NCO in PU. The cryogenic (77 K) mechanical properties of MWCNTs-NH_2_ modified EP-based composites increased up to 0.2 wt% MWCNTs-NH_2_ and then decreased with higher MWCNTs-NH_2_ content. For 0.2 wt% MWCNTs-NH_2_ modified EP-based composites, the tensile strength, flexural strength, impact strength and fracture toughness at 77 K were enhanced by 31.2%, 70.2%, 100.0% and 26.0%, respectively, compared with those of neat EP. The existence of reactive groups (–NH_2_) in MWCNTs-NH_2_, the best dispersibility of MWCNTs-NH_2_ (0.2%) in the EP matrix and the thermal contraction of the EP matrix can help in strengthening the interfacial interaction between MWCNTs-NH_2_ and EP matrix at cryogenic condition, resulting in an effective load transfer. As shown in Figure 4c,d, at cryogenic temperature, the fracture surface of CNTs modified epoxy is much rougher than epoxy and exhibits micromesh cracks. The addition of CNTs increased the resistance to crack propagation in epoxy. Hence, the toughness of the thermoset polymer is improved.

Similarly, the oxidized multi-walled carbon nanotubes (O-CNTs) were added to the epoxy using a reactive oligomer to enhance the cryogenic (77 K) mechanical properties of EP-based composites by Yi et al. [39]. In this research, the EP modified with 15 wt% oligomers containing 0.5 wt% of O-CNTs showed 69.5% improvements in impact strength at 77 K, compared to the unmodified EP. Kim et al. found that the crack resistance and the fracture toughness of carbon fiber reinforced epoxy-based composites at cryogenic temperature (123 K) could also be enhanced by adding the aminated carbon nanotubes into the resin matrix [60]. The aminated CNTs were conducted by chemical surface treatment with triethylenetetramine (TETA). Except for aminated CNTs, the carboxyl (COOH) functionalized CNTs could also improve composites’ mechanical properties. Prusty, et al. reported that the flexural strength of carboxyl (COOH) functionalized CNTs modified EP-based composites increased by 16.2% at 198 K, compared with pristine carbon nanotube reinforced epoxy-based composites [61]. Takeda et al. [62] found that the simultaneous addition of 0.5 wt% CNTs and 10 phr n-butyl glycidyl ether (BGE) lead to the increase in the tension–tension fatigue lives for fiber reinforced bisphenol-F epoxy composites at 77 K.The improvement in fatigue lives can be attributed to the fact that the fatigue life of composites is significantly affected by the matrix material because the introduction of CNTs and BGE resulted in the obviously enhanced glass fiber/epoxy interfacial adhesion [63].

#### 2.1.2. Graphene

Graphene, a new two-dimensional carbon nanomaterial, known for its single-layered atom-thick flatbed structure, has brought a new dimension to the nanotechnology world due to its high thermal conductivity, electrical conductivity and mechanical stiffness (130 GPa) [64,65]. Graphene possesses a higher aspect ratio than CNTs, predominantly ideal for reinforcement. Compared with other nanofillers, the presence of graphene effectively prevented crack propagation by producing a large amount of plastic deformation [66,67,68,69], making it a promising candidate for enhancing the cryogenic properties of thermoset polymer. It has been reported that the uniform dispersion of nanofillers such as graphene and CNTs into polymers plays a significant role in determining the mechanical, thermal, and physical properties of the resultant nanocomposites and is one of the challenging works [68,70]. It is also well confirmed that graphene functionalization could improve the dispersion of graphene in the polymer [71,72].

**Figure 5:**
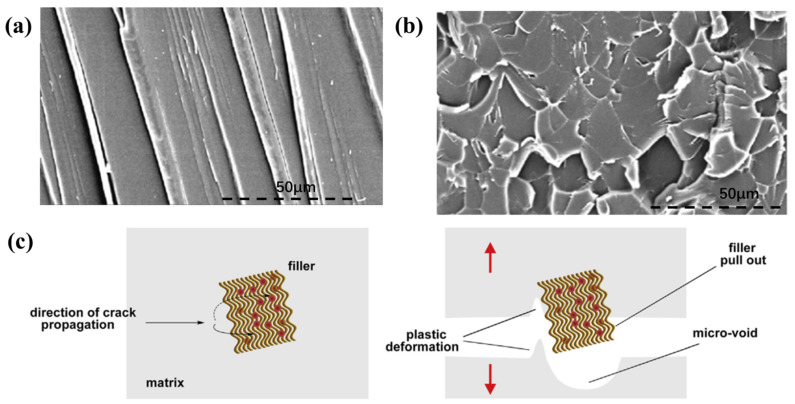
(**a**,**b**) SEM images of the impact fracture surface at 77 K of pure epoxy and graphene/epoxy composites containing 0.1 wt% graphene. Reused with permission from Ref. [40]. Copyright 2012 Elsevier. (**c**) Schematic of the fracture mechanism of graphene/epoxy composites. Reused with permission from Ref. [41]. Copyright 2017 Elsevier.

Shen et al. demonstrated that the introduction of graphene oxide sheets at a proper content of 0.1 wt% into the EP matrix could simultaneously enhance the cryogenic (77 K) tensile strength, Young’s modulus, and impact strength [40]. The tensile and impact strengths of graphene/EP composite reached the maximum with an improvement of 10.5% and 23.7% at 77 K, respectively. At the relatively low content, the graphene with a good dispersion can resist the propagation of cracks in the polymer. Thus, the fracture surfaces of the graphene-modified EP are rougher than those of pure EP (Figure 5a,b). Hussein et al. synthesized a hybrid filler based on graphene oxide/poly p-phenylenediamine (GO-PDA) [41]. At 153 K, the GO-PDA nanofiller could improve the strength, fracture strain, and toughness of EP when added at a low content. Lee et al. added functionalized graphene sheets (FGs) to the epoxy resin to enhance its cryogenic performance [35]. FGS/EP nanocomposites’ strength and toughness increased by 200% and 700% at 143 K, respectively, at the loading of 1.6 wt% FGS. And the CTE of polymer resin reduced about 25% at the same contents. A Fe_3_O_4_ modified graphene oxide (Fe_3_O_4_/GO) nanofiller, which was synthesized by a modified co-precipitation reaction between Fe^2+^ and Fe^3+^ ions in the presence of GO, was used to modify the cryogenic mechanical properties of the polymer by He et al. [44]. The study reflected the CTE of Fe_3_O_4_/GO (0.5 wt.%) modified EP composite is decreased by 51.6% compared with neat EP. Besides, compared to CF/EP composite, the micro-cracks density of Fe_3_O_4_/GO modified CF/EP composite at 77 K is decreased by 60.0%. Recently, Huang et al. compared two different approaches employing GO into CF/EP composites. One is dispersing GO into EP matrix, the other is coating it onto the surface of CF [45]. The result demonstrated that two ways could improve the mechanical properties of composites at cryogenic temperature. However, the latter is a more desirable employment method in the fabrication of GO-modified polymer-based composites for the strong chemical interaction and mechanical interlocking between CF and polymer. At 77 K, the bending strength and modulus of GO coating CF reinforced composites increased by 47.62% and 23.28%, respectively, compared with the controlled sample. The above researches all confirmed that the toughening mechanisms of graphene are crack deflection and plastic void growth due to the improved interface strength caused by polymer/filler CTE mismatch (Figure 5c) at cryogenic temperature.

#### 2.1.3. Other Inorganic Nanomaterials

Besides graphene and CNTs, there are other inorganic nanoparticles used to improve the mechanical properties of polymeric composites, such as silica and nanoclay [26,27,73,74]. Low cost, ease of fabrication and excellent compatibility with polymer have made them more readily available than many other kinds of nanofillers like CNTs and graphene [74,75,76,77,78].

**Figure 6:**
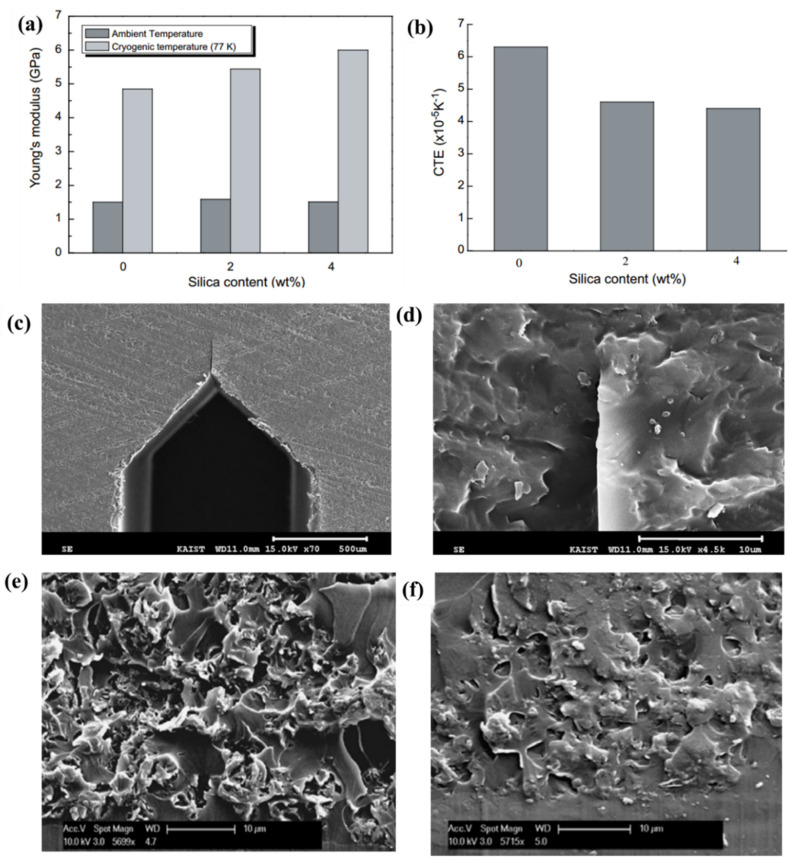
(**a**)The effect of silica content on Young’s modulus of modified polymer composites and (**b**) the average CTE of modified polymer composites from 298 K to 77 K. Reused with permission from Ref. [45]. Copyright 2018 Elsevier. (**c**) the single edge notch bend specimen with a very sharp pre-crack, (**d**) magnified crack tip, fracture surfaces of the specimens tested nanoclay reinforced epoxy at (**e**) 273 Kand (**f**) 123 K. Reused with permission from Ref. [83]. Copyright 2008 Elsevier.

Huang et al. found that the tensile strength, tensile fracture strain of SiO_2_/EP nanocomposites could be increased by almost 42% and 44% respectively by adding 4 wt% SiO_2_ to the diglycidyl ether of bisphenol-F (DGEBF) type EP [45]. With the increase of silicate content, Young’s modulus at 77 K of the SiO_2_/EP nanocomposite increased and the average CTE from 298 K to 77 K decreased (Figure 6a,b). The SiO_2_/EP nanocomposite was prepared by using DGEBF type EP and tetraethylorthosilicate via the sol-gel process. Besides, nano-silica can also be added to EP resin with the direct blending method. This method is more convenient, practical, and economical. By directly adding SiO_2_ nanoparticles into the epoxy matrix, the contact forces and energy absorption capacities of fiber-reinforced composites are improved during the low-velocity impact (LVI) test at a cryogenic temperature [79]. However, as the nano filler itself usually has a strong tendency to agglomerate, adding the filler into the EP directly will result in the non-uniform dispersion of nanofiller and reduce affinity between the filler and the matrix [71]. Wang studied the dispersion of silica modified by different silane coupling agents (SCAs) in DGEBA type EP resin and tested the mechanical properties of these different silica/EP nanocomposites at cryogenic temperature (77 K) [47]. Compared with other coupling agents, the KH560-modified silica enhanced EP nanocomposite has superior performance at cryogenic temperature.

Layered silicate, such as nanoclay, becomes a more promising nanofiller in EP-based nanocomposites due to the high intercalation chemistry, high aspect ratio, thermal stability and low cost. It has been shown in the Unmanned Space Vehicle (USV)-CRYOTANK project of the Italian Aerospace Research Program that the organo-modified nanoclays and layered hydroxides could improve the barrier properties of cyanate-esters and EP based polymer matrix composites [80]. The research conducted by NASA Glenn Research Center has demonstrated that adding a layered silicate clay to an appropriate matrix can significantly improve the performance of a polymer matrix composite for cryogenic tank applications [81]. The nanoclay enhanced EP nanocomposites with 70% lower hydrogen permeability than the base EP resin. Due to a clamping force induced on nanoclay by negative thermal expansion, the lap joint shear strength of nanoclay (1 wt%) modified EP adhesive increased by 8.6% at 77 K [82]. Nevertheless, researchers investigated the mode I fracture toughness (K_IC_) of EP at cryogenic temperature (123 K), which was determined by the 3-point bending method with the single edge notch bend specimens with a very sharp pre-crack (Figure 6c,d). The results revealed the K_IC_ of the nanoclay reinforced EP was lower than pristine epoxy, although the fracture surface was much rougher than that of the pristine EP (Figure 6e,f) [83]. Similar to silica, the challenge of adding nanoclay to polymer matrix is dispersion. Hu found that the incorporation of a block copolymer polyethyleneoxide-co-polypropylene oxide-co-polyethylene oxide (PEO-PPO-PEO) into nanoclay modified EP could improve the dispersion of clay in the matrix and prevent clay agglomerations [46]. Moreover, the tensile strength and Young’s modulus at 77 K of this nanoclay modified hybrid nanocomposite were enhanced by 31.0% and 14.6%, respectively.

The deformation of layered nanoclay under stress could form microvoids and inhibit crack propagation, which does favor to the improvement of the toughness of nanoclay modified polymer-based composites. Besides, the tearing effect by the flake shape of nanoclay is also a fact that increased the toughness of nanoclay modified polymer matrix composites. The transverse cracks in CF reinforced nanoclay-EP composites under cryogenic cycling were significantly reduced when nanoparticle fillers were incorporated at 5 wt%, which was much lower than those used for traditional fillers [84].

### 2.2. Enhancing the Cryogenic Performance of Thermoset Polymer by Introducing the Soft Organic Modifiers

Soft modifiers, such as thermoplastic polymers, rubber, hyperbranched polymer and siloxane also play an important role in improving the toughness of polymer matrix composites [27,28,85]. When adding such soft modifiers into the brittle networks of the thermoset matrix, the flexible chains or groups of soft modifiers contribute to the intermolecular force and stress relaxation at the crack tip in the matrix [18,86], resulting in the improvement in the fracture toughness of thermoset polymer. At cryogenic temperature, the resin contracts, and the introduction of soft modifiers increase the occupied volume of the resin chain. Thus, the toughness would be enhanced [87].

#### 2.2.1. Thermoplastic Polymer

Toughening the thermoset matrix with thermoplastic polymers, especially engineering thermoplastics, has received much attention because it generally does not cause dramatic decrease to other mechanical or physical properties [88,89,90,91].

**Figure 7:**
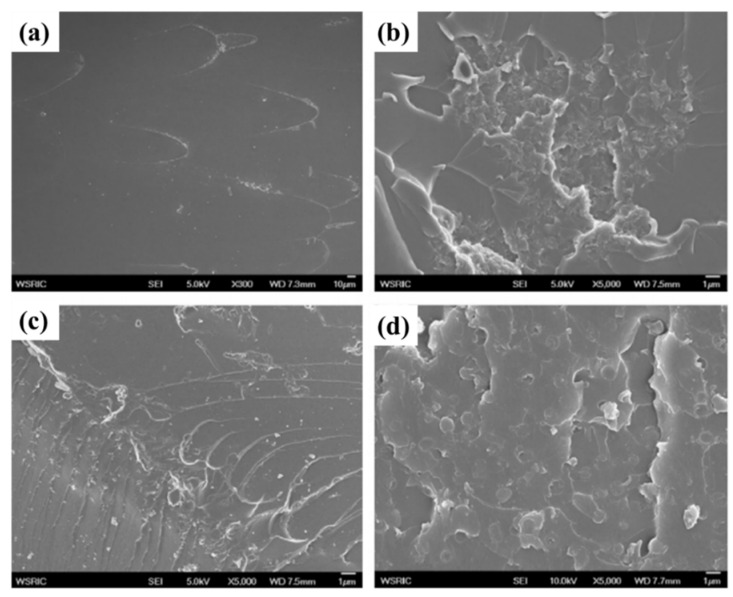
The SEM images of fractured surfaces under impact test at 77 K of (**a**) neat epoxy, (**b**) PBT modified epoxy, (**c**) PC modified epoxy and (**d**) PEI modified epoxy. Reused with permission from Ref. [49]. Copyright 2013 Elsevier.

Owing to the high thermal stability and easy processability, polyethersulfone (PES), was used as an additive into DGEBF type epoxy to enhance the cryogenic mechanical properties by Yang et al. [48]. They found that the tensile strength, tensile fracture strain, and impact strength at 77 K increased with the PES content increasing up to 20 phr. When the content of PES was 20 phr, the remarkable improvement of the mechanical properties might be ascribed to the co-continuous phase structure of PES and EP with excellent interfacial adhesion, which was beneficial to absorb the crack energy and able to shear yielding against the crack. While further increasing the ratio of PES content to 25 phr in the blend, the disconnected EP particles would be surrounded by the continuous phase of PES, which was phase inversion structure. Thus, the reduction of tensile strength, elongation at break, and impact strength occurred. The cryogenic performance of EP can also be improved by using other thermoplastic resins. During the research conduct by He et al., three different types of thermoplastics, polyetherimide (PEI), polycarbonate (PC), and polybutylene terephthalate (PBT) were used to modify EP for cryogenic applications [49]. The impact strength of the modified EP at cryogenic temperature increased with increasing thermoplastic content up to 1.5 wt% and then decreased for further loading (2.0 wt%). As shown in Figure 7, unlike PBT and PEI, the fracture surface of PC modified EP at 77 K reveals a smooth surface with elongated material. It seems that the PC has good bonding with epoxy [92]. The addition of thermoplastic could lower the thermal stress presented in the fiber–EP laminate and improve the micro-crack resistance of fiber-reinforced modified EP composites. They also found that the toughing efficiency of PEI and PC were better than PBT on EP. Wu et al. evaluated the cryogenic temperature (77 K) mechanical properties of DEGBA type EP composites modified by two Kinds of hydroxyl-terminated polyurethanes (HTPU) with different molecular weights [50]. By changing the HTPU content into the epoxy, two-phase structures of homogeneous phase and sea-island structures were successfully obtained. The homogeneous phase resulted in higher enhancing effects on impact and tensile properties, while the sea-island structure showed better retention in the mixture’s glass transition temperature. Li synthesized the EP-functionalized polysiloxane (EPSE) and blended it into bisphenol-F epoxy resin to improve the cryogenic mechanical properties of EP [51]. The EPSE has better compatibility with EP resin because of the existence of an epoxy group in EPSE. The O-Si-O group in the EPSE could enhance the movability of epoxy networks and has better plastic deformation deflecting the crack propagation. Thus, the tensile strength, failure strain, fracture toughness and impact strength at 90 K increased by 11.2%, 33.8%,30.7% and 14.8% respectively, when mixed 10 wt% PSE into DGEBF type EP. Briefly, the toughening of thermoplastic is caused by the resistance to crack propagation and dissipation of energy.

#### 2.2.2. Rubber

Rubber modification as a major toughening approach to overcome the inherent brittleness and high notch sensitivity limit of thermoset polymers has been adopted by many researchers [93,94,95,96]. While the addition of rubber also resulted in reductions of both the glass transition temperatures and the interlaminar shear strength of the fiber-reinforced composites [28,97,98].

**Figure 8:**
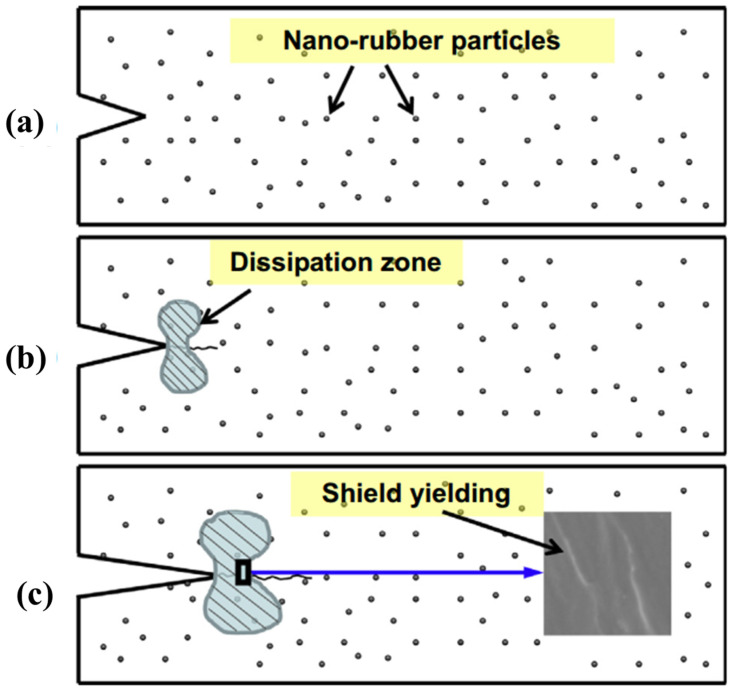
Schematic diagram of the stages of crack propagation in NR particles modified epoxy resins: (**a**) the notched sample, (**b**) the crack extends, (**c**) the crack propagates future. Reused with permission from Ref. [52]. Copyright 2013 Elsevier.

Preformed rubber particles, core-shell rubber (DP5031, Zeon Chemicals Inc., Shanghai, China), and solid carboxyl functional rubber (Nipol1472, Zeon Chemicals Inc.) were used to modify the resin by Nobelen [99]. Their study focused on the influence of the toughener types on the microcracking response to cryogenic cycling of the fiber-reinforced laminates. Compared to the CF/EP composites toughened with preformed rubber particles or core-shell rubber, the carboxyl-functionalized rubber could present homogeneously in the continuous epoxy phase resulting in a dramatic reduction in the crack density of the laminate when exposed to cryogenic cycling. However, when the DP 5045 and DP 5031 rubber particles were combined to modify the resin matrix, a significant drop in the microcrack density was observed. It can be concluded that the concentration of rubber additive is a more critical factor than the type. They also discussed the influence of carboxyl-terminated butadiene acrylonitrile (CTBN) liquid rubbers on the microcracking response of polymer-based composite materials to cryogenic cycling [100]. In their work, it was also shown that an increase of rubber concentration in CF/EP laminates led to a decrease in the microcrack density which resulting from cryogenic cycling. Similarly, the tensile strength, failure strain and fracture toughness (K_IC_) at 77 K of the EP composites were simultaneously enhanced by the addition of Carboxylic nitrile-butadiene nano-rubber (NR) particles [52]. Such mechanical properties at 77 K were observed to increase with the increase of the NR content up to 15 phr. Compared with pure EP, at 77 K, the tensile strength, fracture toughness and failure strain of NR EP blend with 15 phr NR was increased by 40.2%, 48.3% and 55.9%, respectively. The observed increase in mechanical performance could be attributed to the shear yielding deformation of the dissipation zone among the homogeneously dispersed soft NR in the EP matrix brings about the reduction of stress concentration near the crack tip as shown in Figure 8. Nevertheless, Young’s modulus of EP resins was slightly decreased with the increase of the NR particles.

### 2.3. Enhancing the Cryogenic Performance of Thermoset Polymer by Decreasing the Crosslink Density

It is known that the different network structures cause different properties even if the polymers have the same chemical structure. Due to the chemical reaction, the crosslinking network of thermoset polymer resins is usually rigid and brittle. Thus, controlling the network structures of thermoset polymer is also an effective way to obtain the desired properties [101,102,103,104]. Many studies have concluded that the toughness of thermoset EP resin could be enhanced by an alteration of the network topology without using other toughening agents.

Nishijima introduced free volume and free space, which was defined as the unoccupied space between and within the molecules, to explain the toughening mechanism of EP at a cryogenic temperature [105]. The free space of resin networks can be changed by adding plasticizer, altering the molecular weights, the length of chains, or both. From the fracture toughness test results at LHe temperature (2 K), the EP with different crosslinking densities was preferable for cryogenic use [106]. Besides, they used DGEBA as a modifier to improve the fracture toughness of the multifunctional EP tetraglycidyl meta-xylenediamine (TGMXDA) resin system at a cryogenic temperature [87]. The results suggested that epoxies with larger molecular weights among crosslink relaxed stress at the crack tip, even at cryogenic temperatures. By comparing the mechanical and thermal properties of EP systems with different chemical structures, network structures and morphology, Ukei found that the two-dimensional network structured epoxies (phenoxy) were promising because they showed high fracture toughness at cryogenic temperatures [107].

Except for changing the structure of epoxies, the crosslinking network structure of the resin system also can be controlled by changing the types and dosages of curing agents, adding compounds with active groups or changing the curing process. Wang studied the cryogenic performance of the phosphorus-containing bisphenol an epoxy resins/aromatic amine systems. The crosslink density of resin systems was controlled by changing the EP/amine equivalent ratio (SR) [108]. According to this research, the tensile strength, strain energy and impact toughness of the phosphorus-containing bisphenol A EP resin/aromatic amine system reached the maximum when the SR was 0.8. It is known that the internal stress within the bulk material can significantly affect the performance of the finished product by creating micro-cracks and voids. Conversely, the introduction of soft segments into brittle EP resin can dramatically reduce internal stress. Yang et al. introduced flexible diamines (D-230 and D-400) and hyperbranched polymer(H30) into the diethyl toluene diamine (DETD) and Methyltetrahydrophthalic anhydride (MeTHPA) cured DGEBA resin systems respectively and investigated the mechanical properties of the modified EP resin systems at 77 K [54,109]. The results show that the tensile strength and impact strength at 77 K are simultaneously improved by adding flexible diamines and hyperbranched polymer at an appropriate content. Similarly, the addition of other compounds with the active groups, such as n-butyl glycidyl ether [110], polyethylene glycol [111] and EP-functionalized polysiloxane [112], could improve the cryogenic performance of DGEBF or DGEBA based EP. Generally, by decreasing the crosslink density or introducing a soft segment to the brittle network of thermoset polymers, the tensile strength and toughness of the polymer would decrease and increase respectively. Meanwhile, at cryogenic temperature, the internal stress could be reduced. These results suggest that the mechanical properties of thermoset polymers at cryogenic temperature could be enhanced by balancing the internal stress and strength reduction.

## 3. The Research Development of Thermoplastic Polymer for Cryogenic Application

The development of thermoplastic resins, especially high-performance engineerings plastics, such as polyamide (PA), polyetheretherketone (PEEK), polyethersulfone (PES), and perfluoroalkoxy (PFA), promises several advantages over thermoset resins in terms of their improved range of mechanical properties and processing techniques available [34,113,114]. The thermoplastic resins exhibit several times increases in fracture toughness over epoxies, which show great potential for cryogenic applications. There is almost no storage problem with thermoplastic as they can be stored indefinitely at room temperature without deleterious effects [115,116]. In general, thermoplastic polymer-based products are often fabricated using conventional molding methods, such as injection-molding, rotational-molding, extrusion, and compression molding. However, it is not suitable for manufacturing large composites structures for the limitation of processing technologies. With the development of prepreg technology and novel processing techniques such as automated tape laying (ATL) [34,117,118,119,120], it is possible to make large thermoplastic composites structures and composites tanks [5,7]. Although thermoplastics have attracted researchers’ attention for their excellent mechanical properties, lack of a curing stage, less hazardous chemical compositions and improved recycling convenience, the reports about the cryogenic performance of thermoplastics and their composites are rare. Hence, in this section, the research and application development on the cryogenic performance of neat thermoplastic and modified thermoplastic polymeric are discussed, respectively. Despite there are many challenges for cryogenic application, we think the research on the cryogenic performance of thermoplastic polymer and its composites will become a meaningful work in the future.

### 3.1. The Cryogenic Performance of Pristine Thermoplastic Polymer

Among all kinds of thermoplastic polymers, the high-performance engineering thermoplastic polymers are widely applied in various extreme environments for their excellent strength, fracture toughness, weatherability and processability. To date, the reports about the cryogenic performance of thermoplastic and their composites are mainly focused on the high-performance thermoplastic polymer.

**Figure 9:**
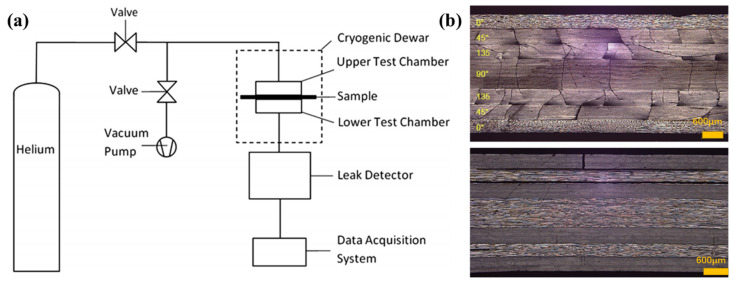
(**a**) Schematic of the test setup used for permeability testing of composites. Reused with permission from Ref. [125]. Copyright 2017 Elsevier. (**b**) Cryogenically induced high density microcracking and delaminations in quasi-isotropic laminate (**top**) and large-scale delamination and single microcrack in cross-ply laminate (**bottom**). Reused with permission from Ref. [126]. Copyright 2014 Elsevier.

In 1990, Ahlborn et al. studied the mechanical performance of CF reinforced PPEK and PC composites at cryogenic temperature (5 K and 77 K) [121]. The result demonstrated that the tensile strength and interlaminar shear strength of CF/PEEK composites are better than those of CF/PC composites. Although the interlaminar shear strength of the CF/PC composites was lower, it still performed better than conventional EP-based composites. Besides, the author concluded that the high cryogenic fracture strain of some advanced thermoplastics was beneficial for the tensile fracture strength of fiber-reinforced polymer composites. PEEK has been demonstrated to have superior mechanical properties, wear resistance, chemical resistance, and flame-retardant property, which has attracted some deep researches on the cryogenic performance of PEEK-based composites [122]. In 2001, NASA supported a project that involved the fabrication of a PEEK composite cryogenic fuel line for use on future reusable launch vehicles [123]. Fabrication of the fuel line was done with ADC Acquisition Company’s proprietary in-situ thermoplastic fiber placement equipment. Flanagan et al. investigated the permeability of four different CF/PEEK composites manufactured using autoclave after cryogenic cycling [124]. The results show that the permeability of CF/PEEK composites ($2.3\sim 5.2\times 10^{-17}\mathrm{mol}/\mathrm{smPa}$) manufactured by the autoclave process was low enough to make it a suitable material choice for cryogenic storage. The schematic of the test setup used for the permeability of composites is shown in Figure 9a. For all samples, cryogenic cycling had little effect on the leak rate of CF/PEEK composites unless cycling caused micro-cracks of the matrix. After cryogenic cycling treatment, the quasi-isotropic laminates ([0_4_/45_4_/135_4_/90_4_]_s_) suffered extensive micro-cracks and short delaminations (Figure 9b) and the cross-ply laminates([0_4_/90_4_]_2s_) displayed a number of extensive inter-ply delaminations [125]. Grogan et al. [126] studied the damage formation and mechanical properties of automated tape-laid CF/PEEK composites under cryogenic temperature. The damage (void and inclusion content) of tape-laid CF/PEEK laminates were measured using 3D X-ray computed tomography (CT). Compared with the autoclave process, the tape-laid material exhibited lower strength values, particularly in the matrix-dominated directions, which is similar to the reference [124]. Based on the test results and a novel numerical methodology (extended finite element method), they developed a sub-model based on extended finite element method–cohesive zone methodology (XFEM-CZM) to predict intra- and inter-ply damage in an internally pressurized cryogenic tank. The results demonstrated that the average crack densities of the plies on the inner surface were higher than their corresponding plies on the outer surface of the tank. Besides, the thermal and mechanical performance of fiber-reinforced thermoplastic polyimide composites at cryogenic temperature was studied by Karen et al. [127]. Interestingly, compared to aging in an unloaded condition, the strength and stiffness of fiber reinforced thermoplastic polyimide composites will increase regardless of laminate lay-up, when aging the material under a constant strain condition at cryogenic temperature. Li et al. examined the tensile and flexural properties of PES at 77 K to discuss the application prospect of PES in the cryogenic engineering field [128,129]. The findings suggested that the reinforced structure, prepared methods and load condition can make a significant difference in the cryogenic performance of thermoplastic polymer composites.

The fatigue performance of polymers can make differences in their service life when they exposed to cryogenic and load environment for a long time. Hartwig et al. [130] discussed the fatigue lives of PEEK and PC under different stress ratio *R* at 77 K. The results demonstrate that the fatigue lives of PEEK and PC decreased when increasing the stress ration. As shown in the formula 1, under a given stress amplitude (*S_a_*), the mean stress (*S_m_*) increases with the stress ratio.

(1) $$S_{m}=S_{a}\left(1+R\right)/\left(1-R\right)$$

The rise in mean stress indicates that the tensile part of cyclic loading increase, which would promote the crack initiation and propagation and then decrease the fatigues lives. However, they also found that the fatigue endurance limits of PEEK-based composites were lower than EP-based composites. Takeda et al. [131] investigated the stress intensity factors for several crack configurations in glass/epoxy composites with under tension at cryogenic temperatures. The mechanical mean stress combined with the thermal load magnifies the stress intensity factor. The correlation between stress intensity factor and fatigue crack growth is a powerful tool for fail–safe design approaches applied to lightweight structures [132]. In general, the higher stress intensity factor, the greater the possibility to fracture [133,134].

In addition to mechanical properties, manufacturing costs should be taken into ac-count. The application of high-performance thermoplastic polymers is very costly for its high price and relatively complicated manufacturing process. Relatively cheaper thermoplastic materials could be an alternative solution if their performance at cryogenic temperatures is found to be satisfactory. Weiss investigated the thermal conductivity, expansion and mechanical strength under cryogen temperature (77 K and 4 K) of acrylonitrile-butadiene-styrene copolymer (ABS) specimens that were processed by 3D printing technology [135]. Compared with EP and high-performance thermoplastic polymer, the cryogenic mechanical properties of ABS are lower. This research provides some important data to evaluate their application prospects in the cryogenic temperature regime. Veltin investigated the performance of fully recyclable, lightweight, low-cost, thermoplastic polypropylene (PP) composite tapes at low temperatures and discussed the applicability of these materials for Liquefied Natural Gas (LNG) applications [136]. At cryogenic temperature, the microcracks between the resin and the fibers due to the CTE difference is a topic of concern. The author introduced the PP-based Single Polymer Composites (SPCs) which contain matrix and reinforcement of the same chemical composition to minimize that issue since the constituent components of the laminates possess the same CTE. The PP-based composites have a skin-core-skin structure where the skin is a PP copolymer and the core is a PP homopolymer. The tensile strength (77 K) of the [0/90] and [±45] laminates is 268 MPa and 37 MPa, respectively, and the corresponding failure strains are 3.9% and 6.5%. Furthermore, during the cryogenic low-velocity impact tests on all-PP tubes, the indentation as large as 64% of the inner diameter generated during the low velocity test at 4.88 m/s is fully recovered which demonstrated the viscoelastic nature of the PP at such low temperature. Although the tensile and bending strength of PP-based composites at cryogenic temperature is lower than CF/EP or CF/PEEK composites, it is hopeful to design a structure operating at cryogenic conditions that have for its sufficient ductility and lower cost.

### 3.2. The Cryogenic Performance of Modified Thermoplastic Polymer

**Figure 10:**
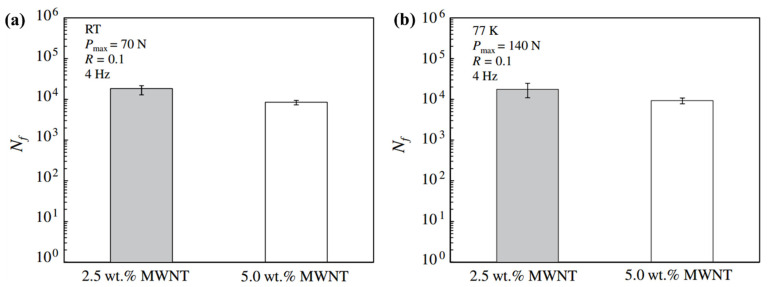
Fatigue lives of CNTS/polycarbonate composites at (**a**) room temperature and (**b**) 77 K. Reused with permission from Ref. [139]. Copyright 2014 Elsevier.

Different from thermoset polymers, the main method to improve the mechanical, thermal or electrical properties of thermoplastic is the physical modification method. The introduction of nanomaterial into the polymer is an effective way to enhance its thermal and mechanical properties. Some efforts have been made to improve the cryogenic performance of thermoplastic polymer and its composites by adding a low loading level of nanofillers, such as CNTs, GO and short carbon fibers (SCFs) [128,129,137,138,139,140]. Takeda et al. studied the cryogenic tensile response of CNT-reinforced polycarbonate composites and used the finite element simulations method to predict the elastic modulus and the stress state within the composites [137,138]. The Young’s modulus and 0.2% offset yield strength at 77 K increased with increasing CNTs-contents. However, with the CNTs contents increased, at 77 K, the ultimate tensile strength and fracture strain of CNTs-reinforced polycarbonate composites increased and then decreased. And the tensile strength (152 MPa) and fracture stain (5.18%) reached the maximum at 1.7 vol% CNTs. However, compared with the neat polycarbonate, the enhanced effectiveness in cryogenic mechanical property is limited. Wei et al. [139] and Takeda et al. [138] also investigated the flexural and tensile fatigue response of CNTs reinforce polycarbonate composites. As these research demonstrated the 2.5 wt% CNTs/polycarbonate composite at 77 K and room temperature had a longer fatigue life than the 5.0 wt% CNTs/polycarbonate composite under the same applied load. The addition of CNTs could improve the fatigue resistance and fatigue lives of polycarbonate at 77 K. The local existence of CNTs agglomerates in 5.0 wt% CNTs/polycarbonate composite led to the poor mechanical properties. Therefore, the fatigue properties of the 5.0 wt% CNTs/polycarbonate composite is lower than 2.5 wt% CNTs/polycarbonate composite. Meanwhile, they also found an interesting phenomenon that although the maximum applied load at 77 K is two times higher than that at room temperature, the fatigue lives of the composites at 77 K are similar to those at room temperature (Figure 10). Bahçeli et al. [140] studied the cryogenic properties of CNTs reinforced Ultra-high molecular weight polyethylene (UHMWPE) Laminated Composites. The CNTs were added to UHMWPE by two different methods, one is by placing CNTs in the form of buckypaper (CNT BP) during manufacturing, and the other is by producing CNTs reinforced high density PE films (CNT/HDPE) through the melt mixing process. Place all of these CNTs based films were into UHMWPE piles and then fabricate different CNTs based film enhanced UHMWPE composites via the hot-pressing technology. Although both types of films could enhance the interlaminar shear strength of UHMWPE composites at room temperature, only the CNTs/HDPE modified UHMWPE composites showed 3% increase at 77 K. The addition of CNT BP into UHMWPE has shown none improvement in interlaminar shear strength at 77 K.

**Figure 11:**
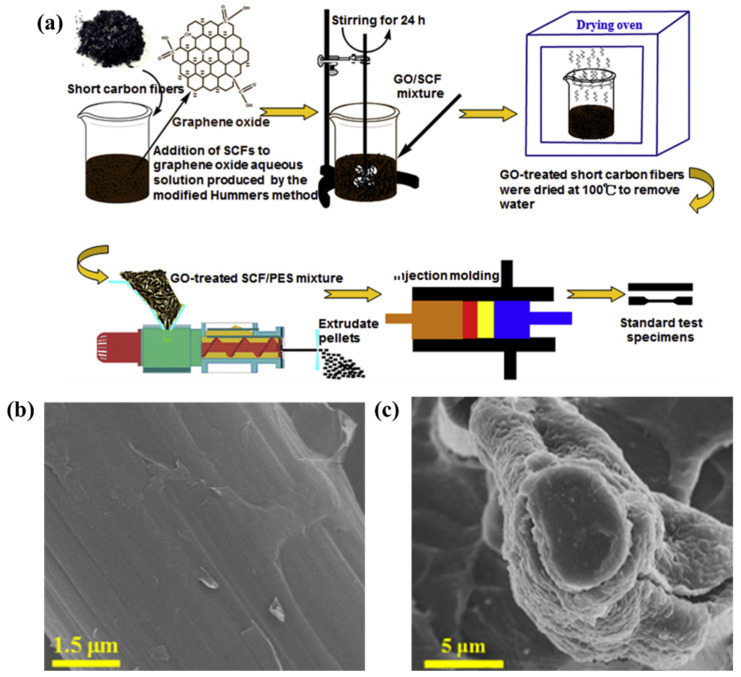
(**a**) Schematic of GO sizing onto short carbon fibers and the process chart of preparing GO-coated SCFs composite specimens. Reused with permission from Ref. [128]. Copyright 2015 Elsevier. (**b**) The SEM image of the GO-coated carbon fibers with the 0.5 wt% GO, (**c**) The SEM images of the tensile failure surfaces of 0.5 wt% GO-coated SCF/PES composite at 77 K. Reused with permission from Ref. [129]. Copyright 2016 Elsevier.

LI et al. [128,129] introduced GO, short carbon fibers (SCFs), both GO and SCFs simultaneously, and GO-coated SCFs into PES to get PES-based composites via the extrusion and injection molding techniques. The preparation process of GO-coated SCFs and its based PES composites specimens was shown in Figure 11a. Subsequently, the mechanical properties of PES based composites at 77 K were examined. They concluded that the GO-coated SCF/PES composites greatly enhanced cryogenic mechanical properties with the highest values compared to other PES composites. At 77 K, the tensile and flexural strength of 0.5 wt% GO-coated SCF/PES composite reached almost 223 MPa and 415 MPa. This phenomenon could be explained by the high interfacial bonding between GO, GO-coated SCF and PES (Figure 11b,c).

## 4. The Research Development of the Polymer for Liquid Oxygen Application

### 4.1. The Acceptance-Testing Method of Materials Used in the Liquid Oxygen Environment

**Figure 12:**
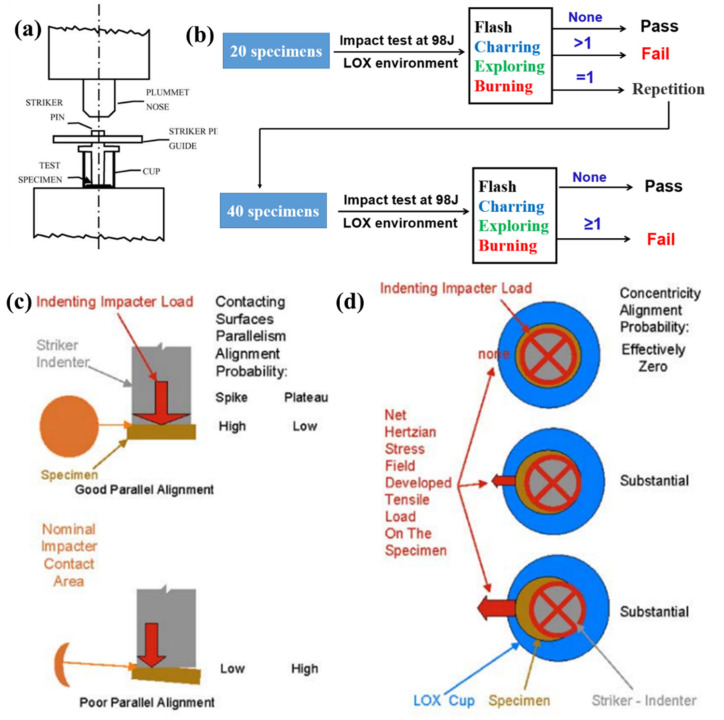
(**a**) the plummet pin assembly detail of impact test. Reused with permission from Ref. [147]. Copyright 2006 Elsevier. (**b**) the acceptance criteria of testing material for use in the liquid oxygen system. (**c**) the contact conditions of the specimen with different surfaces, (**d**) the possible relative positions of cup, specimen and striker during the mechanical impact test. Reused with permission from Ref. [144]. Copyright 2006 American Society of Testing Materials.

To the polymeric composites applied for LOX, except to meet the mechanical requirements, the composites used in LOX must also be chemically compatible with LOX. [5,6,7] Compatibility with LOX is a relative quality determined by how readily and to what degree a material will react with oxygen [141]. The chemical mechanism of the LOX compatibility of polymers has been confirmed to be oxidation reactions. It is common Knowledge that many materials burn or explode in the presence of oxygen when exposed to external stimuli such as mechanical impact, crash, friction, or static electricity [142]. Materials that are impact-sensitive with liquid oxygen are generally also sensitive to the reaction by other forms of energy in the presence of oxygen. Meanwhile, the mechanical impact test is easy to conduct and the impact energy can also be controlled. So the impact test is adopted widely by researchers to characterize the compatibility of the material with LOX [17,143]. Since the 1950s, this test method has resulted in a large amount of data. The test equipment, parameters and steps were standardized (ASTM D2512, ASTM G86) [9,17,142]. According to ASTM test standard, the specimen of the material is placed at the bottom of an aluminum cup filled with LOX, a flat-tipped steel pin resting on the specimen, and then a 10 Kg plummet drop from 1 m height to strike the steel pin(Figure 12a) [142,144,145,146,147]. During the test, any explosion, burning, noticeable ash, or charring of specimen is considered as a reaction. As shown in Figure 12b, if there are no reactions in 20 separate specimens, the specimen could be used at the LOX environment. If only one reaction occurs in 20 separate specimens, 40 more samples are needed to test. NO reactions happen in these 40 specimens, this material has the ability to be used in the LOX environment. One or more reactions occur, the material cannot be used in the LOX environment. It has been concluded that it is a strict “pass/fail” test that material must meet if they are to be used in the LOX system. Based on the test standard (ASTM D2512-95), Gerzeski proposed a cascade of probabilistic mechanisms that teams Hertzian fracture and Kinetic friction to study the mechanical impact in LOX ignition threshold sensitivity testing of polymer and polymer composites [144]. A portion of the mechanical impact energy is transformed into thermal energy and a local hot spot formed in the specimen. The local temperature of the specimen could be increased by hundreds of degrees Celsius in a short time (1μs or less) due to the thermal energy that transformed from mechanical impact energy [145,146]. During the mechanical impact test, the contract surface condition between specimen and striker and the relative position of specimen and striker may both affect the test result (Figure 12c,d) [144]. Wang et al. used a commercial finite element analysis tool to analyze the transient fracture mode and energy conversion process of thermoset polymer during the impact test (ASTM D2512) qualitatively [47]. The results demonstrated that about 25% of the Kinetic energy (98J) was transformed into sliding energy and the rest was transformed into internal energy.

### 4.2. Research Progress in Liquid Oxygen Compatibility of Polymers

In the beginning, researchers mainly focused on the LOX impact sensitivity of some commercial organic material. In 1959, Clippinger and Morris [148] reported the detailed impact Sensitivity test results of some common organic materials on exposure to LOX conducted by the Martin Company’s Baltimore Division for the first time. According to the report, except for the Teflon, almost all organic materials were impacted sensitively, such as synthetic elastomers, cellulose silicone, silicate-based oils, nylons, thermo-setting phenolics and epoxies. Hoggatt studied the Liquid oxygen impact sensitivity of Mylar, Kapton and Fluorinated ethylene propylene (FEP) coated Kapton polymeric film samples. They confirmed that plain Kapton and FEP films were LOX compatible [149]. In 1967, Clark et al. reviewed the compatibility of structural materials (metals and nonmetals) with oxygen [141]. According to their report, nickel and copper alloys, stainless steels, aluminum alloys, polytetrafluoroethylene (TFE) and polychlorotrifluoroethylene (CTFE) are available for LOX use. Robinson et al. tested fiber-reinforced polymer composites compatibility with LOX [142]. The polymers included EP, bismaleimide, polyimide, phenolic, cyanate ester, PEEK, and fluorinated epoxy. While none of these polymer-based composites could meet the standard mechanical impact test (ASTM D2512-95). Wang et al. investigated the LOX compatibility of EP and cyanate ester co-cured compounds, and confirmed that improving the anti-oxidation properties of the polymer could enhance its LOX compatibility [147]. From the above reports, it can be concluded that the better flame retardancy, the fewer reactions of material occurred during the liquid oxygen impact sensitivity test.

It has been confirmed that the higher impact energy would result in a rapid increase of temperature at the local surface of the specimen to form a hot spot. With the rapid increase of temperature, a lot of relatively high activity radicals such as hydroxyl radicals will be released on the surface of the specimen and even cause local thermal degradation of the polymer. The high activity radicals can cause the incessant chain reaction. Thus, the reactions (charring, noticeable ash, explosion, burning) of organic materials under the impact test will appear [150,151,152]. Wu et al. analyzed the surface element compositions of polyphenylene sulfide specimens before and after impact tests at LOX environment [150]. They found that the oxygen contents of the specimen with reaction (flash) during the mechanical impact were higher than the specimen without flash. Consequently, it may be concluded that increasing the polymer flame retardancy or thermal stability or both will improve the LOX compatibility of the polymer [147,148,149]. Therefore, improving the polymer’s flame retardancy or thermal stability or both through physical or chemical modifications seems to be a possible way to meet the LOX environment application requirements.

Table 2 lists some modified polymer systems reported in the literature that get through the mechanical impact LOX compatibility test. The detailed information of such material as shown below.

**Figure 13:**
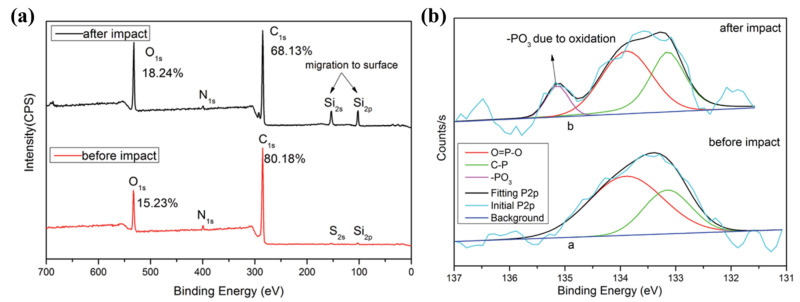
(**a**) the XPS spectra and (**b**) P2p peak fitting curves of the specimen before and after mechanical impact under the liquid oxygen environment. Reused with permission from Ref. [159]. Copyright 2016 Royal Society of Chemistry.

Wu et al. incorporated 9,10-dihydro-9-oxa-10-phosphaphenanthrene-10-oxide (DOPO) [151] and 10-(2,5-dihydroxyphenyl)-9,10-dihydro-9-oxa-10-phosphaphenanthrene-10-oxide (ODOPB) [152] into EP and evaluated its LOX compatibility. The DOPO and ODOPB are two kinds of phosphorus-containing reactive flame retardants with diphenyl structure which has high thermal stability and good oxidation resistance. In this way, the LOX compatibility EP was synthesized successfully. During the impact test, the modified EP would generate phosphoric oxyacid and a lot of phosphoric-oxygenic free radicals. The existence of the acid shields the material with LOX, preventing the oxidation and decomposition of the epoxy resin. The phosphoric-oxygenic free radicals could capture the highly active free radicals such as hydrogen and hydroxyl radicals and then constrain the chain reaction. From the previously published results, the type of EP and cure agent also make an important effect on the LOX compatibility of the modified resin system [153,154,155]. Wu et al. reported that the liquid oxygen compatibility of the DOPO modified DGEBA resin system was better than the DOPO modified DGEBF resin system under the same condition [153]. Wu et al. also discussed the LOX compatibility of bromine-modified DGEBA resin curing with different curing agents (DDM and DDS) [154,155]. According to the impact test results, the modified EP resin curing with DDM showed good compatibility with LOX than curing with DDS [155]. Peng et al. introduced a DOPO-based trisiloxane containing phosphorus and silicon compounds into the EP molecular structure to enhance the LOX compatibility of EP resin [156]. By analyzing the surface elements of the specimens before and after mechanical impact in the LOX environment through the XPS method, the Si2p and Si2s signal peaks appeared obviously in the overall XPS spectra (Figure 13a). Meanwhile, based on the XPS test result, the PO_3_ group in the pyrophosphate and polyphosphate generated during the mechanical impact was also demonstrated (Figure 13b). The existence of Si and P elements breaks the chain reactions caused by oxidation reaction during the mechanical impact at LOX environment, hence improve the LOX compatibility of the polymer. The functioned nanomaterial could improve the flame retardancy, thermal stability and cryogenic mechanical properties of the resin system. Mixing a certain amount of the DOPO modified nanofiller (boehmite) into pure epoxy could also improve the liquid oxygen compatibility of the resin system [157]. Peng et al. synthesized a phosphorous-containing benzoxazine monomer (PBA), and treated PBA as harder to cure EP [158]. They found that the EP/benzoxazine included PBA copolymer could pass the impact test (ASTM D2512-95). Due to the flame-retardant ability of phosphorous and the thermal stability of benzoxazine [159,160], the modified polymer mentioned above showed great potential for manufacturing the LOX composites tank for its comprehensive performance (mechanical properties and LOX compatibility). Therefore, it has been proven that improving the flame retardancy, thermal stability of polymer is an effective way to enhance its LOX compatibility [150,151,152,153,154,155,156,157,158].

## 5. Summary and Perspectives

In this review, we have summarized the research development of polymer for cryogenic application. In general, compared with room temperature, at cryogenic temperature, the strength, modulus and fracture toughness (K_IC_) of polymers increased. While the impact resistance, tensile fracture strain of polymer decreased dramatically. Accordingly, researchers utilized various methods to enhance the polymer ductility and reduce the thermal stress of polymeric composites. As these reports demonstrated, these modification methods are useful to enhance the cryogenic performance of polymer and polymer-based composites. According to these studies, some functionalized polymers showing great potential for cryogenic application, such as CNTs/EP, GO/EP and phosphoric EP and son on. Meanwhile, for the polymer used in the LOX environment, improving the flame retardancy, thermal stability and toughness of the polymer is an effective way to enhance its LOX compatibility. Despite significant achievements in this field, some directions expect to be explored shortly.

1. Compared with thermosetting or general used thermoplastic polymers, the high-performance engineering thermoplastic polymers have more potential for cryogenic application due to their prominent comprehensive properties (strength, toughness, flame retardancy and recyclable performance). However, the research of high-performance engineering plastic for cryogenic use are seldomly reported. With the development of energy and aerospace, more attention should be paid on the study of high-performance engineering thermoplastic and its composites for cryogenic application.
2. The damage evolution and fracture mechanism and the LOX compatibility mechanism of polymer and its composites at cryogenic temperature need to be studied further. A deep understanding of the cryogenic performance of polymer and its composites would help researchers to find more effective ways to obtain suitable material.
3. During the cryogenic performance test process of the material, several cryogenic media such as LHe, LN_2_, and LOX are necessary to provide the cryogenic test environment. Because these cryogenic liquid fluids are extremely volatile, it would take a lot of time and liquids to test the cryogenic performance of the material. Thus, it will be meaningful and interesting to find out the relationship between room temperature performance and cryogenic temperature performance of polymer and its composites or construct the prediction model based on the data of the report to predict the cryogenic performance polymer.
4. The present reports mainly focused on the effectiveness of the methods to improve the cryogenic performance such as mechanical properties, LOX compatibility and impermeability of polymer and its composites. However, the processability of the modified polymer such as viscosity and pot life, which are essential for practical applications, is not included. Future work is required to provide greater insight into the processability of material.

## Figures and Tables

**Table 1:** Cryogenic mechanical properties of modified thermoset polymers.

Resin System ^1^	Modifier ^1^	Content	Temperature	Mechanical Properties	Ref.
DGEBF/DETDA	MWCNTS	0.5 wt%	77 K	Tensile strength: 119.35 MPa Tensile modulus: 5.26 GPa Tensile fracture strain: 3.20% Impact strength: 35.56 KJ/m^2^	[37]
DGEBA/EP-PU/DETDA	MWCNTS-NH_2_	0.2 wt%	77 K	Tensile strength: 120 MPa Tensile modulus: 7.0 GPa Flexural strength: 275 MPa Flexural modulus: 8.0 GPa Impact strength: 32 KJ/m^2^ Fracture toughness: 3.25 MPa·m^1/2^	[38]
DGEBA/15 wt% Oligomer/D-230	O-CNTs	0.5 wt%	77 K	Tensile strength: 91.7 MPa Tensile modulus: 3.6 GPa Impact strength: 46 KJ/m^2^	[39]
DEGBA/DETDA	Graphene	0.1 wt%	77 K	Tensile strength: 140.0 MPa Tensile modulus: 7.0 GPa Impact strength: 21.9 KJ/m^2^	[40]
DGEBA/DETA	GO-PDA	1 wt%	150 K	Tensile strength: 70 MPa Tensile modulus: 3.29 GPa Tensile fracture strain: 2.50% G_IC_: 666.32 J/m^2^ K_IC_: 1.58 MN/m3/2	[41]
DGEBA/diluent/D-230	FGSs	1.6 wt%	143 K	Tensile strength: 82.91 MPa Tensile modulus: 5.54 GPa Tensile fracture strain: 1.65%	[42]
DGEBA/TETA	GO	0.2 wt%	193 K	Tensile strength: 150 MPa Tensile modulus: 4.7 GPa Tensile fracture strain: 3.7%	[43]
DGEBA/D-230	Fe_3_O_4_/GO	0.5 wt%	77 K	Tensile strength: 96.4 MPa Tensile modulus: 5.9 GPa Tensile fracture strain: 19.5% Impact strength: 44.2 MPa K_IC_: 2.1 MN/m^3/2^	[44]
DGEBF/DETD	SiO_2_	4 wt%	77 K	Tensile strength: 77.63 MPa Tensile modulus: 4.62 GPa Tensile fracture strain: 2.2%	[45]
DGEBA/BCE/BCP	Nano-clay	3 wt%	77 K	Tensile strength: 169 MPa Tensile modulus: 5.5 GPa Tensile fracture strain: 2.2%	[46]
DGEBA/DDM	KH560-modified silica	1 wt%	90 K	Tensile strength: 169 MPa Tensile modulus: 9.0 GPa Tensile fracture strain: 2.5%	[47]
DGEBF/DETD	PES	20 phr	77 K	Tensile strength: 86.64 MPa Tensile modulus: 4.32 GPa Tensile fracture strain: 2.87%	[48]
DGEBA/D-230	PBT/PEI/PC	1.5 wt%	77 K	Impact strength: 34.5 KJ/m^2^(PBT) Impact strength: 39.8 KJ/m^2^(PEI) Impact strength: 36.01 KJ/m^2^(PC)	[49]
DGEBA/DETDA	HTPU	5 phr	77 K	Tensile strength: 161.67 MPa Tensile modulus: 6.89 GPa Tensile fracture strain: 2.58% Impact strength: 30 KJ/m^2^	[50]
DGEBF/DDM	EPSE	5 wt%	90 K	Tensile strength: 210 MPa Tensile fracture strain: 3%	[51]
DGEBF/DDM	EPSE	5–8 wt%	77 K	K_IC_: 2.35 MN/m3/2 (5 wt%) K_IC_: 2.6 MN/m3/2 (8 wt%)	[52]
DGEBF/DETD	NR	15 phr	77 K	Tensile strength: 129.98 MPa Tensile modulus: 4.13 GPa Tensile fracture strain: 3.15% K_IC_: 2.2 MN/m^3/2^	[53]
DGEBA/NA/A-178	HK021	80 phr	77 K	LSS: 9.7Mpa	[54]
DGEBF/MeTHPA	H30	10 wt%	77 K	Tensile strength: 115.6 MPa Tensile modulus: 3.8 GPa Tensile fracture strain: 3.1% Impact strength: 32 KJ/m^2^	[55]

^1^ Abbreviations: DGEBF, diglycidyl ether of bisphenol-F; DETD, diethyl toluene diamine; MWCNTs, Multi walled carbon nanotubes; DGEBA, diglycidyl ether of bisphenol-A;EP-PU, epoxy grafted polyurethane; MWCNTS-NH2, aminated Multi walled carbon nanotubes; D-230, polyoxypropylenediamine; O-CNTs, oxidized multi-walled carbon nanotubes; GO-PDA, graphene oxide/poly p-phenylenediamine;FGs, functionalized graphene sheets.TETA, Triethylene tetra amine;GO, graphene oxide; BCE, 2,2-bis(4-cyanatophenyl) isopropylidene; BCP, block copolymer; DDM, 4,4′-diaminodiphenylmethane; KH-560, 3-glycidoxypropyltrimethoxysilane; PES, Polyethersulfone; PBT, Polybutylene terephthalate; PEI, Polyether imide; PC, Polycarbonate; HTPU, hydroxyl-terminated polyurethanes; EPSE, epoxy-functionalized polysiloxane;NR, carboxylic nitrile-butadiene nano-rubber; NA, nadictetrahydric-methylanhydride; A-178, commercial name of silane coupling agent; HK021, commercial name of diol molecule; MeTHPA, Methyltetrahydrophthalic anhydride; H30, commercial name of hyperbranched polyester.

**Table 2:** The cryogenic performance of the reported materials that has the potential ability for liquid oxygen system use.

Resin System ^1^	Modifier ^1^	Contents	Thermal Stability	Mechanical Properties	Ref.
DGEBA/DOPO/DDS	DOPO	7.7 phr	T(10%): 381 °C	-	[141]
DGEBA/ODOPB/DDM	ODOPB	18.6 phr	T(5%): 393 °C T(50%): 531 °C	Tensile strength: 148.9 MPa (90 K) Tensile fracture strain:2.27% (90 K) K_IC_ = 1.58 MN/m^3/2^ (77 K)	[152]
DGEBA/TBBPA/DDM	TBBPA	20.5 phr	T(5%): 365 °C	Tensile strength:86.37 MPa (77 K) K_IC_ = 1.63 MN/m^3/2^ (77 K)	[154]
DGEBA/AlOOH−DOPO−GPTS/ DDM	AlOOH−DOPO−GPTS	20 wt%	T(5%): 367.8 °C T(10%): 380.3 °C	Tensile strength: 126 MPa (90 K) Tensile fracture strain:1.57% (90 K)	[157]
EP/PBA/BA	PBA/BA	24 wt%/34.3 wt%	T(5%): 351.2 °C T(50%): 533.7 °C	-	[158]

^1^ Abbreviations:DOPO, 9,10-dihydro-9-oxa-10-phosphaphenanthrene-10-oxide;DDS, 4,4-Diaminobisphenol sulfone; ODOPB, 10-(2,5-dihydroxyphenyl)-9,10-dihydro-9-oxa-10-phosphaphenanthrene-10-oxide; TBBPA, Tetrabromobisphenol A; AlOOH, boehmite; GPTS, 3-glycidoxypropyltrimethoxysilane;PBA, phosphorous-containing benzoxazine monomer;BA, bisphenol A benzoxazine.

## Data Availability

Data sharing not applicable.
